# Sorafenib attenuates liver fibrosis by triggering hepatic stellate cell ferroptosis via HIF‐1α/SLC7A11 pathway

**DOI:** 10.1111/cpr.13158

**Published:** 2021-11-22

**Authors:** Siyu Yuan, Can Wei, Guofang Liu, Lijun Zhang, Jiahao Li, Lingling Li, Shiyi Cai, Ling Fang

**Affiliations:** ^1^ Department of Pharmacy The First Affiliated Hospital of Anhui Medical University Hefei China; ^2^ Department of Urology The Second People's Hospital of Hefei Hefei China; ^3^ School of Pharmacy Anhui University of Chinese Medicine Hefei China; ^4^ School of Pharmacy Anhui Medical University Hefei China

**Keywords:** ferroptosis, HIF‐1α/SLC7A11, HSCs, liver fibrosis, sorafenib

## Abstract

**Objectives:**

Evidences demonstrate that sorafenib alleviates liver fibrosis via inhibiting HSC activation and ECM accumulation. The underlying mechanism remains unclear. Ferroptosis, a novel programmed cell death, regulates diverse physiological/pathological processes. In this study, we aim to investigate the functional role of HSC ferroptosis in the anti‐fibrotic effect of sorafenib.

**Materials and Methods:**

The effects of sorafenib on HSC ferroptosis and ECM expression were assessed in mouse model of liver fibrosis induced by CCl_4_. In vitro, Fer‐1 and DFO were used to block ferroptosis and then explored the anti‐fibrotic effect of sorafenib by detecting α‐SMA, COL1α1 and fibronectin proteins. Finally, HIF‐1α siRNA, plasmid and stabilizers were applied to assess related signalling pathway.

**Results:**

Sorafenib attenuated liver injury and ECM accumulation in CCl_4_‐induced fibrotic livers, accompanied by reduction of SLC7A11 and GPX4 proteins. In sorafenib‐treated HSC‐T6 cells, ferroptotic events (depletion of SLC7A11, GPX4 and GSH; accumulation iron, ROS and MDA) were discovered. Intriguingly, these ferroptotic events were not appeared in hepatocytes or macrophages. Sorafenib‐elicited HSC ferroptosis and ECM reduction were abrogated by Fer‐1 and DFO. Additionally, both HIF‐1α and SLC7A11 proteins were reduced in sorafenib‐treated HSC‐T6 cells. SLC7A11 was positively regulated by HIF‐1α, inactivation of HIF‐1α/SLC7A11 pathway was required for sorafenib‐induced HSC ferroptosis, and elevation of HIF‐1α could inhibit ferroptosis, ultimately limited the anti‐fibrotic effect.

**Conclusions:**

Sorafenib triggers HSC ferroptosis via HIF‐1α/SLC7A11 signalling, which in turn attenuates liver injury and fibrosis.

## Graphical abstract

Sorafenib triggers hepatic stellate cell ferroptosis by inhibiting the HIF‐1α/SLC7A11 pathway to attenuate liver fibrosis. Treatment with sorafenib induces a decrease of HIF‐1α, which in turn reduces SLC7A11 expression in HSCs. Then leads to GPX4, GSH depletion and ROS excess, and ultimately induces HSC ferroptosis and ECM reduction.

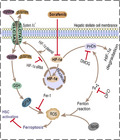

## 1 INTRODUCTION

Approximately 2 million humans died of chronic liver disease per year worldwide.^1^ Liver fibrosis is considered as a reversible pathophysiological process during the progression of chronic liver disease, which is characterized by excessive accumulation of extracellular matrix (ECM).^2^ Hepatic stellate cells (HSCs), the main ECM‐producing cells, cause the deposition of fibrous tissue and scar formation.^3^ In response to liver injury, quiescent HSCs undergo a complex activation process and eventually trans‐differentiate into activated HSCs. Markers of activated HSCs, such as cytoskeletal protein α‐smooth muscle actin (α‐SMA), collagen 1α1 (COL1α1) and fibronectin, are upregulated in this process.^4^ Studies from our own and others laboratories have shown that inhibition of HSC decreases ECM deposition and alleviates liver fibrosis.^5^, ^6^, ^7^

Ferroptosis, a novel type of programmed cell death, is characterized by intracellular iron overload and accumulation of lipid reactive oxygen species (ROS).^8^ The morphology and mechanism of ferroptosis are different from traditional programmed cell death, such as apoptosis, autophagy and necrosis. Morphologically, ferroptotic cells exhibit reduced mitochondrial volume, condensed mitochondrial membrane density, absent mitochondrial cristae and even ruptured outer mitochondrial membranes. Mechanically, ferroptosis is associated iron metabolism disorder, lipid peroxidation accumulation, glutathione (GSH) and solute carrier family 7 member 11 (SLC7A11) deficiency.^9^ Ferroptotic events have been found in HSC cell lines treated with erastin, sorafenib and buthionine sulfoximine, and HSC growth and ECM accumulation were significantly decreased in this progess.^10^, ^11^ Moreover, some traditional Chinese medicines exhibit anti‐fibrotic effects through inducing HSC ferroptosis.^12^, ^13^

Sorafenib, a multiple kinases inhibitor, is well known for its anti‐tumour effect. In addition, sorafenib regulates HSC viability via inhibiting cell proliferation and promoting apoptosis, afterwards, exhibits anti‐fibrosis effect in liver.^14^, ^15^ Recent studies proved sorafenib as a ferroptotic inducer, which triggers a series of ferroptotic events in different kinds of cells.^9^, ^16^, ^17^ Ferroptosis has been observed in sorafenib‐treated HSCs as well.^10^, ^11^ Nevertheless, the underling mechanism remains unclear.

During the progress of fibrosis, regions of hypoxia were observed in liver, accompanied by increased expression of hypoxia‐inducible factor 1α (HIF‐1α).^18^, ^19^ In nonalcoholic fatty liver disease model, knockout HIF‐1α specifically in hepatocyte could reduce liver fibrosis.^20^ Meanwhile, sorafenib decreased HIF‐1α protein level and caused apoptosis of hepatocellular carcinoma cell.^21^ Interestingly, decrease in HIF‐1α with inhibitors or specific siRNA enhanced cell ferroptosis.^22^ As a transcriptional factor, HIF‐1α regulates several gene level including SLC7A11.^23^, ^24^ Knockout of HIF‐1α sharply decreased SLC7A11 protein in rat brain tissue.^25^ So we wondered whether HIF‐1α/SLC7A11 is involved in sorafenib‐induced HSC ferroptosis.

In the study, we find that sorafenib induces ferroptotic events in HSCs via HIF‐1α/SLC7A11 pathway, and HSC ferroptosis is involved with reduction of α‐SMA and CoL1α1. On the contrary, ferroptotic inhibitors, ferrostatin‐1(Fer‐1) and deferoxamine (DFO), dramatically reduce the anti‐fibrotic effect of sorafenib. Intriguingly, sorafenib triggers ferroptosis in HSCs with no effect on hepatocytes or macrophages. Our study may provide a potential therapeutic target for sorafenib in the treatment of liver fibrosis.

## 2 MATERIALS AND METHODS

### 2.1 Mouse model of liver fibrosis and sorafenib treatment

6‐ to 8‐week‐old male C57BL/6 mice were provided by Anhui University of Traditional Chinese Medicine. The animal experimental protocol was approved by the University Animal Care and Use Committee. Mice were randomly divided into five groups (n=6 per group) including the vehicle group, CCl_4_‐treated group, CCl_4_ + sorafenib (2.5, 5, 10 mg/kg)‐treated groups. The groups treated with CCl_4_, CCl_4_ + sorafenib were subjected intraperitoneal injections of olive oil with 10% CCl_4_ (#56‐23–5, MACKLIN, China; 10 ml oil/kg; twice a week) for 8 weeks to induce liver fibrosis. The vehicle group was subjected intraperitoneal injections of the same dose of olive oil. After 4 weeks, each mouse in the groups treated with CCl_4_ + sorafenib was orally administered sorafenib (#sc‐357801A, Santa Cruz, USA) daily. The vehicle group and the CCl_4_‐treated group were orally administered the same dose of saline daily. 8 weeks later, all mice were euthanized; serum and livers were obtained. The serum was used to detect liver function indicators. Livers were paraffin‐embedded or stored at −80°C for further analysis.

### 2.2 ALT/AST/HYP assay

The levels of alanine aminotransferase (ALT), aspartate aminotransferase (AST) and hydroxyproline (HYP) in serum were assessed using ALT Kit (#C009‐2–1, Jiancheng, China), AST Kit (#C010‐2–1, Jiancheng, China) and HYP Kit (#A030‐2–1, Jiancheng, China) according to manufacturer's instructions.

### 2.3 Histopathology and immunohistochemistry staining

Haematoxylin and eosin (H&E), Sirius Red and Masson's trichrome staining were performed for histological studies. Expression of α‐SMA (1:100; #AF1032, Affinity, China), SLC7A11 (1:200; #ab175186, Abcam, UK), GPX4 (1:100; #381958, ZEN‐BIO, China) and PTGS2(1:100; #501253, ZEN‐BIO, China) was detected by immunohistochemistry staining. Stained sections were observed via inverted fluorescence microscope (#OLYMPUSIX83, Tokyo, JPN). Quantitative analysis of Masson's trichrome staining and Sirius red staining was performed with ImageJ software. The stained image was imported into ImageJ software, and the colour threshold was adjusted until the collagen sections (blue areas in the Masson's trichrome‐stained image and red areas in the Sirius red‐stained image) were selected; then, the area was calculated. Afterwards, the entire staining of the tissue was selected to calculate the total area. The quantitative analysis of collagen staining (also known as the volume fraction of collagen) was the ratio of the positive collagen area to the total tissue area.

### 2.4 Cell culture and treatment

Rat HSC line (HSC‐T6) was obtained from the Chinese Academy of Science. HSC‐T6 were cultured in Dulbecco's modified Eagle medium (DMEM; #SH30022.01, HyClone, USA) with 10% foetal bovine serum (FBS; Biological Industries, Israel) and incubated at 37°C with 5% CO_2_. Cells were grown to 70% confluence and then exposed to drugs (sorafenib, Fer‐1 (#S7243, Selleck, USA), DFO (#D9533, Sigma, USA), ZVAD‐FMK (#S7023, Selleck, USA), necrosulfonamide (#S8251, Selleck, USA), DMOG (#S7483, Selleck, USA)) at the indicated concentrations for 24 h. Cells of the control group were grown in drug‐free medium containing equal amounts of DMSO.

### 2.5 Cell viability and cell cytotoxicity analyses

HSC‐T6 cells were seeded into 96‐well plates. When the cell density reached 70%, different concentrations of sorafenib were added, and equal amounts of DMSO without drug were exposed to the control group for 24 h. Cell viability and cell cytotoxicity were assessed with Cell Counting Kit‐8 (CCK‐8; #C0042, Beyotime, China) and LDH Release Assay Kit (#C0017, Beyotime, China) on the basis of the manufacturer's instructions.

### 2.6 Iron/GSH/MDA assay

HSC‐T6 cells were cultured and treated with drugs as described above. Cells are gently collected in PBS solution with a cell spatula. 1% Triton was used to permeabilize the cell membrane, and the cell lysates were centrifuged to obtain. The relative iron, GSH, and malondialdehyde (MDA) concentration in cell lysates were assessed via Iron Assay Kit (#A039‐2–1, Jiancheng, China), Glutathione Assay Kit (#A006‐2–1, Jiancheng, China) and MDA detection Kit (#A003‐4–1, Jiancheng, China) according to the manufacturer's instructions.

### 2.7 ROS assay

Intracytoplasmic ROS were detected by oxidation sensitive fluorescent probe DCFH‐DA (#D6883, Sigma, USA) and analysed by flow cytometry.

### 2.8 Observation of mitochondria morphology in cells

HSC‐T6 cells were inoculated into the cell culture dish, collected with or without sorafenib treatment (the final number of cells was no less than 10^7^) and fixed, dehydrated, macerated and embedded. Embedded blocks were cut into 70 nm‐thick sections and stained with lead citrate. The images were acquired using a transmission electron microscope (TEM; #JEM1400, JEOL, JPN).

### 2.9 Semi‐quantitative reverse transcription‐polymerase chain reaction (qRT‐PCR)

qRT‐PCR assays were performed as described.^6^ Specifically, total RNA was extracted from HSC‐T6 cells using TRIzol reagent (#257401, Invitrogen, USA) and reverse transcribed by cDNA Reverse Transcription Kit (#RR037A, Takara, Japan). Relative quantitation by real‐time PCR was performed using TB Green PCT Master Mix (#RR820L, Takara, Japan). Expression levels of target genes relative to β‐actin were evaluated with 2^−ΔΔCt^ method. The following primer sequences were available**:**

PTGS2 (rat)‐forward: 5′‐TGCTGTTCCAACCCATGTCA‐3′; PTGS2 (rat)‐reversed: 5′‐TGTCAGAAACTCAGGCGTAGT‐3′; PTGS2 (mouse)‐forward: 5′‐GGGAGTCTGGAACATTGTGAA‐3′; PTGS2 (mouse)‐reversed: 5′‐GTGCACATTGATAGTAGGTGGACT‐3′; HIF‐1α (rat)‐forward: 5′‐GGTGCTAACAGATGATGGT‐3′; HIF‐1α (rat)‐reversed: 5′‐CTCGTGTCCTCAGATTCC‐3′.

### 2.10 HIF‐1α RNA interference and HIF‐1α Overexpression analyses

Small short interfering RNAs (siRNAs) targeting HIF‐1α gene sequence (5′‐CCAUGUGACCAUGAGGAAATT‐3′) were designed and synthesized by Biogenetech (China). Plasmid of HIF‐1α *(pCDNA3*.*1*‐*CMV*‐*3flag*‐*EF1*‐*ZsGreen*‐*T2A*‐*Puro)* was designed and synthesized by Hanbio Biotechnology (China). The transfection process was carried out as described previously.^26^ Then, qRT‐PCR was used to determine the efficiency of gene knockout and overexpression.

### 2.11 Immunofluorescence analysis

Immunofluorescence assays were performed as described in our previous description.^6^ Primary antibodies were as follows: HIF‐1α (1:100; #340462, ZEN‐BIO, China) and SLC7A11 (1:200). Secondary antibodies were as follows: Alexa Fluor 405‐conjugated anti‐rabbit IgG (H+L) (1:50; #SA00003‐2, ProteinTech, China). All images were collected with confocal laser scanning microscope (#LSM800, Leica, JPN), and fluorescence intensities of target proteins were calculated using ImageJ software.

### 2.12 Western blot analysis

The total proteins of liver tissues and cells were extracted by RIPA (#P0013C, Beyotime, China) with phosphatase inhibitor and PMSF (#P0012S, Beyotime, China). Immunoblotting was performed as previously described.^6^ Primary antibodies were as follows: α‐SMA (1;500), COL1α1 (1:5000), fibronectin (1:500; #A12932, Abclonal, China), SLC7A11 (1:5000), GPX4 (1:1000), PTGS2 (1:1000), HIF‐1α (1:1000), β‐actin (1:5000; #66009‐1‐lg, ProteinTech, China) and Histone H3 (1:1000; #A2631273, Abclonal, China). Secondary antibodies were as follows: peroxidase‐conjugated anti‐rabbit IgG (#2301, ZSGB‐BIO, China) and peroxidase‐conjugated anti‐mouse IgG (#2305, ZSGB‐BIO, China**)**. The protein samples were visualized using the ECL‐chemiluminescent kit (#WBKLS0100, Millipore, USA) and analysed by ImageJ software.

### 2.13 Statistical analysis

All experiments were performed at least three times, and statistical analysis was performed by using GraphPad Prism 5. Data were presented as mean ± SD, and statistical significance was determined by ANOVA with the post hoc test. All significance levels were set at 0.05.

## 3 RESULTS

### 3.1 Ferroptosis occurs in mouse fibrotic liver induced by sorafenib

**FIGURE 1:**
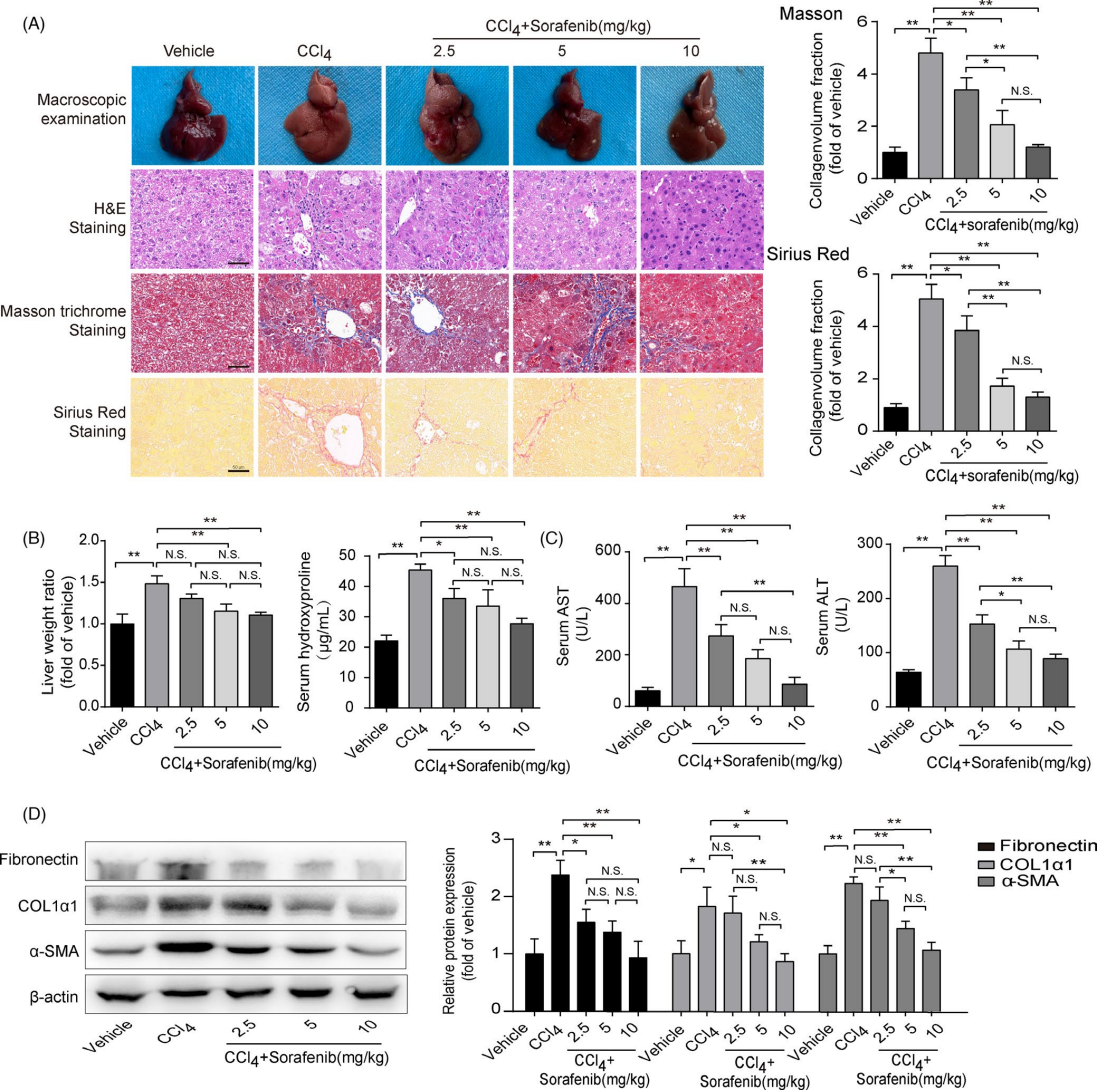
Ferroptosis occurs in mouse fibrotic liver induced by sorafenib. Mouse liver fibrosis model was established by CCl_4_ induction. Different concentrations of sorafenib (2.5, 5, 10mg/kg) were orally administered into the mice. (A) Macroscopic examinations of mouse livers were collected, and liver sections were stained with H&E, Masson's trichrome staining and Sirius Red (Scale bar: 50 μm). Data for Masson's trichrome staining and Sirius red staining were presented as the mean ± SD of 3 independent experiments. *p < 0.05, **p < 0.01. N.S. not significant. (B) The liver/body weight ratio, the levels of HYP, (C) ALT and AST in mouse serum were caculated. Data were presented as the mean ± SD of 3 independent experiments. *p < 0.05, **p < 0.01. N.S. not significant. (D) Western blot analyses of α‐SMA, COL1α1 and fibronectin proteins were performed. (n = 3 in every group). Data were presented as the mean ± SD of 3 independent experiments. *p < 0.05, **p < 0.01. N.S. not significant

It has been proved that sorafenib attenuates liver fibrosis via regulating ECM accumulation and inflammatory reaction.^14^, ^27^ In line with the previous studies, we found that hepatocyte degeneration, inflammatory cell infiltration, fibrous scarring and collagen deposition were all aggravated in the CCl_4_‐treated group, while these changes were remarkably mitigated in sorafenib‐treated groups (Figure 1A). Besides, sorafenib decreased the elevated liver/weight ratio triggered by CCl_4_ (Figure 1B). Moreover, HYP level elevated in the CCl_4_‐treated group, which was significantly reduced by sorafenib (Figure 1B). Sorafenib also significantly reduced the serum levels of ALT and AST (Figure 1C). Fibrotic markers, α‐SMA, COL1α1 and fibronectin, were overexpressed in the CCl_4_‐treated group, while were notably reduced in sorafenib‐treated groups (Figure 1D).

Above data confirm that sorafenib alleviates CCl_4_‐induced liver injury and fibrosis. To further explore the underlying mechanism, ferroptotic markers, GPX4, SLC7A11 and PTGS2, were stained in the lobes of livers. As illustrated in Figure S1, these markers were barely expressed in the vehicle group. GPX4 and SLC7A11 immunostaining signals were obviously observed in HSCs around the fibrotic scars induced by CCl_4_, whereas sorafenib treatment reduced GPX4 and SLC7A11 expressions (Figure S1). It is worth mentioning that GPX4 and SLC7A11 proteins were also significantly increased in liver parenchymal after CCl_4_ induction (Figure S1). Additionally, PTGS2 signals in HSCs were enhanced in sorafenib‐treated liver compared to fibrotic mouse liver (Figure S1). α‐SMA immunostaining signals were abated in sorafenib‐treated livers (Figure S1). Overall, these data suggest that sorafenib attenuates mouse liver fibrosis, which is accompanied by the ferroptotic markers in HSCs.

### 3.2 Sorafenib inhibits HSC activation by triggering ferroptosis in vitro

**FIGURE 2:**
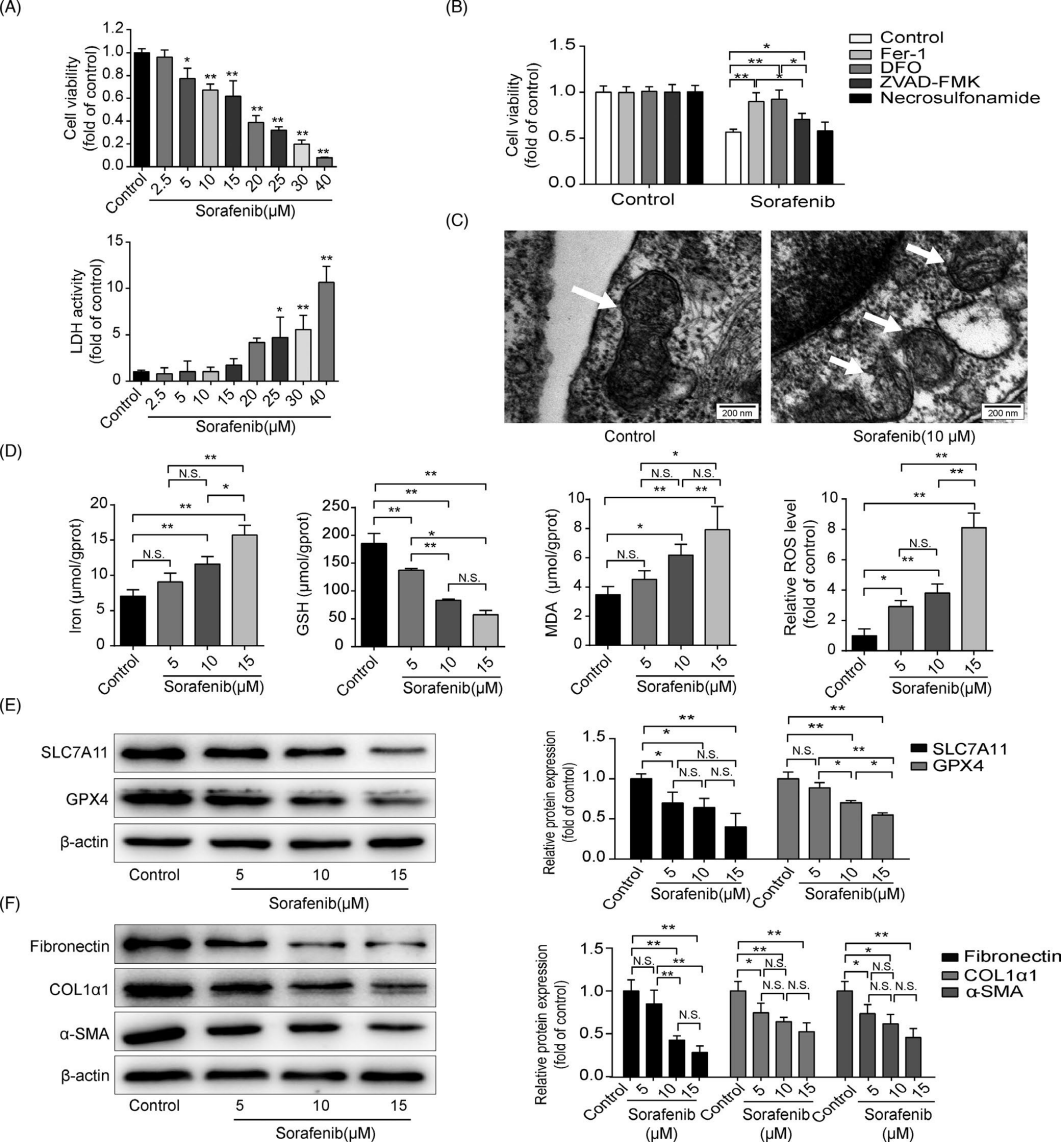
Sorafenib inhibits HSC activation by triggering ferroptosis in vitro. (A) Cell viability of HSC‐T6 cells with different concentrations of sorafenib for 24 h was detected by CCK‐8. Cytotoxicity of AML‐12 cells was assayed by LDH Kit. Data were presented as the mean ±SD of 3 independent experiments. *p < 0.05, **p < 0.01. (B) Fer‐1 (1 μM), DFO (100 μM), ZVAD‐FMK (10 μM) or necrosulfonamide (0.5 μM) were exposed to HSC‐T6 cells with or without of sorafenib (10 μM), and cell viability was assayed by CCK‐8. Data were presented as the mean ± SD of 3 independent experiments. *p < 0.05, **p < 0.01. (C) Mitochondria in control and sorafenib‐treated groups were observed by transmission electron microscope (Scale bar: 200 nm). HSC‐T6 cells were treated with sorafenib (5, 10, 15 μM) for 24 h. (D) Iron release, MDA content and GSH expression in cell lysates were detected by kits. Intracellular ROS generation was detected with DCFH‐DA probe. Data were presented as the mean ± SD of 3 independent experiments. *p < 0.05, **p < 0.01. N.S. not significant. (E) Western blot analyses of SLC7A11 and GPX4 proteins were performed. Data were presented as the mean ± SD of 3 independent experiments. *p < 0.05, **p < 0.01. N.S. not significant. (F) Western blot analyses of α‐SMA, COL1α1 and fibronectin proteins were performed. Data were presented as the mean ± SD of 3 independent experiments. *p < 0.05, **p < 0.01. N.S. not significant

Given that HSCs as the main contributor of ECM production, HSC‐T6 cells were cultured with different concentrations of sorafenib for 24 h. As displayed in Figure 2A, HSC‐T6 cell viability was dose‐dependently inhibited by sorafenib at concentrations ranging from 5 to 40 μM. Meanwhile, there was no toxicity in hepatocyte (mouse, AML‐12 cell line) exposure to sorafenib not more than 20 μM (Figure 2A). Therefore, sorafenib at the dose of 5, 10 and 15 μM was used in the following experiments. Intriguingly, sorafenib‐mediated growth inhibition in HSC‐T6 cells was totally interrupted by Fer‐1 and DFO (Figure 2B). ZVAD‐FMK, an apoptotic inhibitor, also impeded sorafenib inhibition effect in some degree (Figure 2B). However, necrosulfonamide, an inhibitor of necroptosis, had no effect on the cell viability (Figure 2B). Both ferroptotic inhibitors and apoptotic inhibitor hampered the suppression of cell viability in sorafenib‐treated HSC‐T6 cells, Fer‐1 and DFO exhibited a higher ability to reverse the inhibition of HSC viability than that of ZVAD‐FMK (Figure 2B). Additionally, mitochondrial condensation and rupture were observed in the sorafenib‐treated HSC‐T6 cells (Figure 2C). Ferroptotic events were also found in sorafenib‐treated HSC‐T6 cells, which were evidenced by decreased GSH content, increased iron level and lipid peroxidation products (Figure 2D). Protein levels of GPX4 and SLC7A11 were markedly decreased in sorafenib‐treated HSCs (Figure 2E). Additionally, the levels of α‐SMA, COL1α1 and fibronectin were down‐regulated by sorafenib compared with the control group (Figure 2F). In conclusion, above data suggest that ferroptosis is involved in sorafenib‐induced HSC inhibition and ECM reduction.

### 3.3 Sorafenib triggers HSC ferroptosis with no effect on hepatocytes or macrophages

**FIGURE 3:**
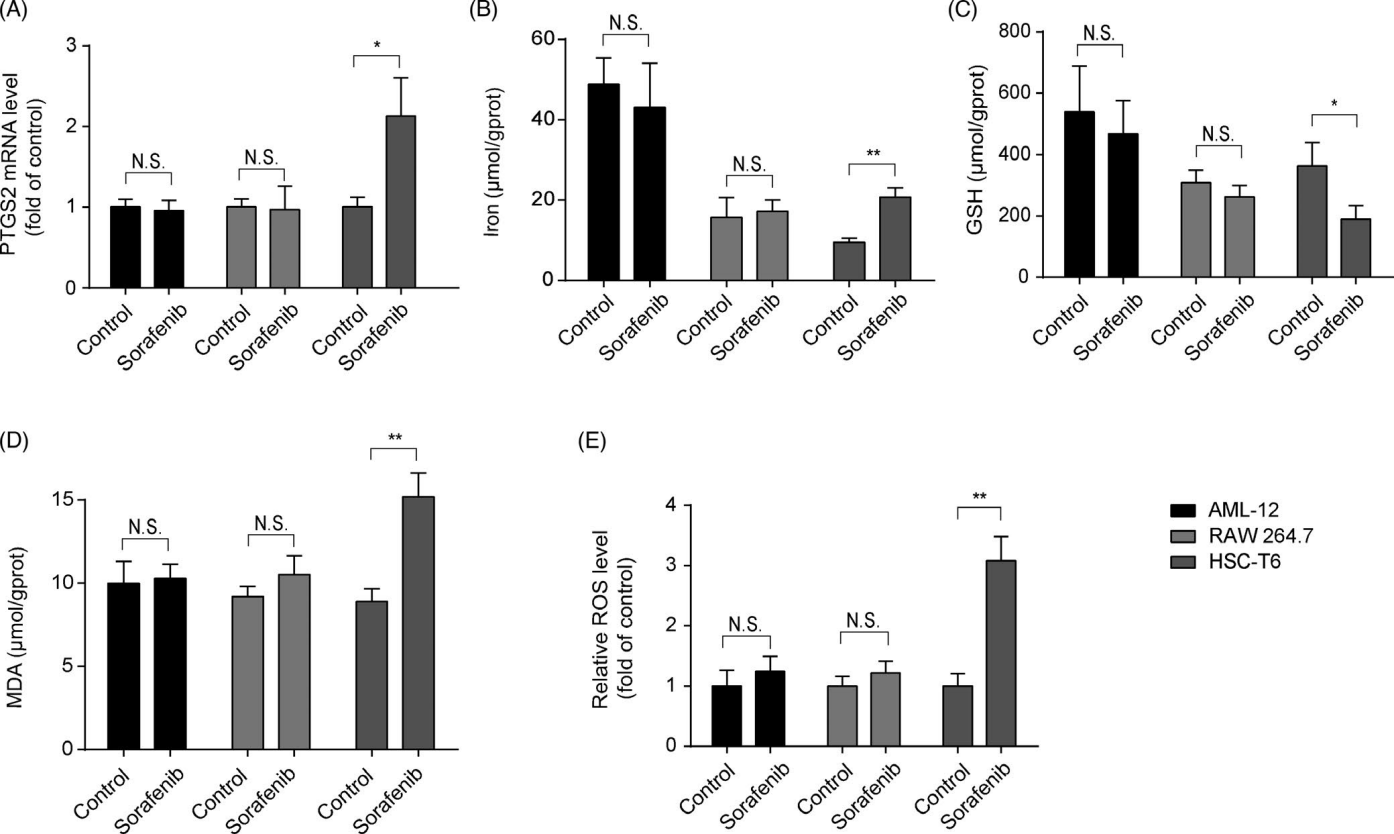
Sorafenib triggers HSC ferroptosis with no effect on hepatocytes or macrophages. AML‐12, RAW 264.7 and HSC‐T6 cells were treated with sorafenib (10 μM) for 24 h. (A) The mRNA level of PTGS2 was assayed by qRT‐PCR. The contents of (B) iron, (C) GSH and (D) MDA in cell lysates were measured by kits. Data were presented as the mean ±SD of 3 independent experiments. *p < 0.05, **p < 0.01. N.S. not significant. (E) Intracellular ROS generation was detected with DCFH‐DA probe. Data were presented as the mean ± SD of 3 independent experiments. **p < 0.01, N.S. not significant

It is well known that liver is composed of hepatocytes, HSCs, macrophages, etc.^28^ Inducing ferroptosis in activated HSCs rather than other cells, such as hepatocytes and macrophages, is an effective strategy for the treatment of liver fibrosis. Thus, HSC‐T6, AML‐12 and RAW 264.7 cells (mouse macrophage cell line) were cultured with sorafenib for 24 h separately. Levels of PTGS2 mRNA (Figure 3A), iron (Figure 3B), MDA (Figure 3D) and ROS (Figure 3E) were elevated by sorafenib in HSC‐T6 cells, while GSH content (Figure 3C) was declined. However, exposed of AML‐12 and RAW 264.7 cells to sorafenib neither of them showed the ferroptotic events (Figure 3A‐E). Together, the data indicate that sorafenib causes ferroptosis in HSCs with little effect on hepatocytes and macrophages.

### 3.4 Blockade of HSC ferroptosis abolishes sorafenib‐induced anti‐fibrotic effect

**FIGURE 4:**
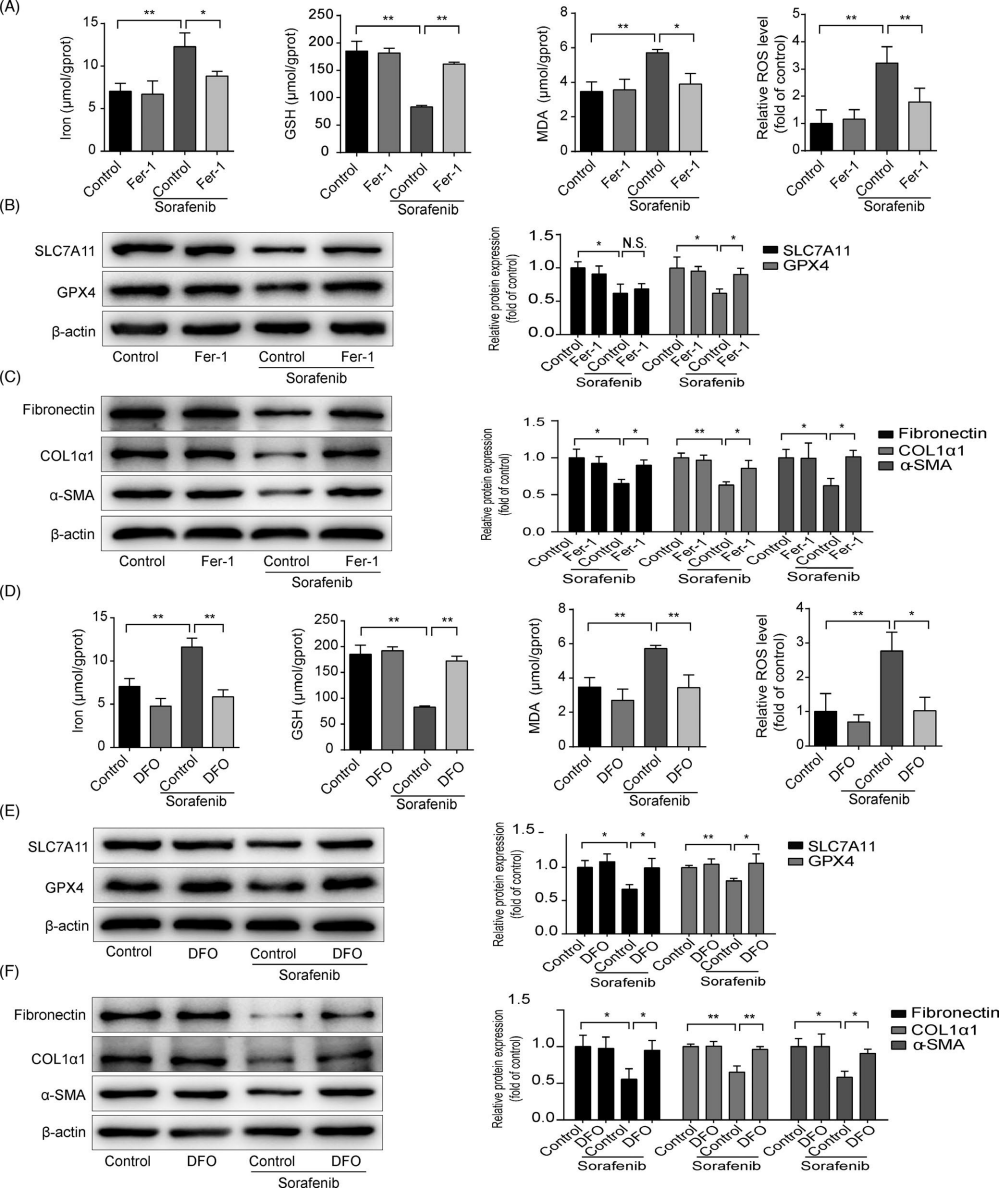
Blockade of HSC ferroptosis abolishes sorafenib‐induced anti‐fibrotic effect. HSC‐T6 cells were exposed to Fer‐1(1 μM), DFO (100 μM) or/and sorafenib (10 μM) for 24 h. (A) and (D) Iron, MDA and GSH contents in cell lysates were measured by kits. Intracellular ROS generation was detected with DCFH‐DA probe. Data were presented as the mean ± SD of 3 independent experiments. *p < 0.05, **p < 0.01. (B) and (E) Western blot analyses of SLC7A11 and GPX4 proteins were performed. Data were presented as the mean ± SD of 3 independent experiments. *p < 0.05, **p < 0.01. N.S. not significant. (C) and (F) Western blot analyses of α‐SMA, COL1α1 and fibronectin proteins were performed. Data were presented as the mean ± SD of 3 independent experiments. *p < 0.05, **p < 0.01

To further investigate the functional role of HSC ferroptosis in the anti‐fibrotic effect of sorafenib, HSC‐T6 cells were exposed to Fer‐1 and DFO for 24 h. As illustrated in Figure 4A and D, both Fer‐1 and DFO could completely abolish sorafenib‐induced ferroptotic events. Meanwhile, reduced GPX4 protein level in sorafenib‐treated HSC‐T6 cells could be reversed by Fer‐1 and DFO (Figure 4B and E), whereas SLC7A11 protein was partially elevated by DFO, but not affected by Fer‐1 (Figure 4B and E). In addition, reduced ECM expression in sorafenib‐treated HSC‐T6 cells was observed, and this process was impaired by Fer‐1 and DFO (Figure 4C and F). In summary, inhibition of HSC ferroptosis impairs sorafenib‐induced anti‐fibrotic efficacy.

### 3.5 HIF‐1α expression is decreased during sorafenib‐induced anti‐fibrosis

**FIGURE 5:**
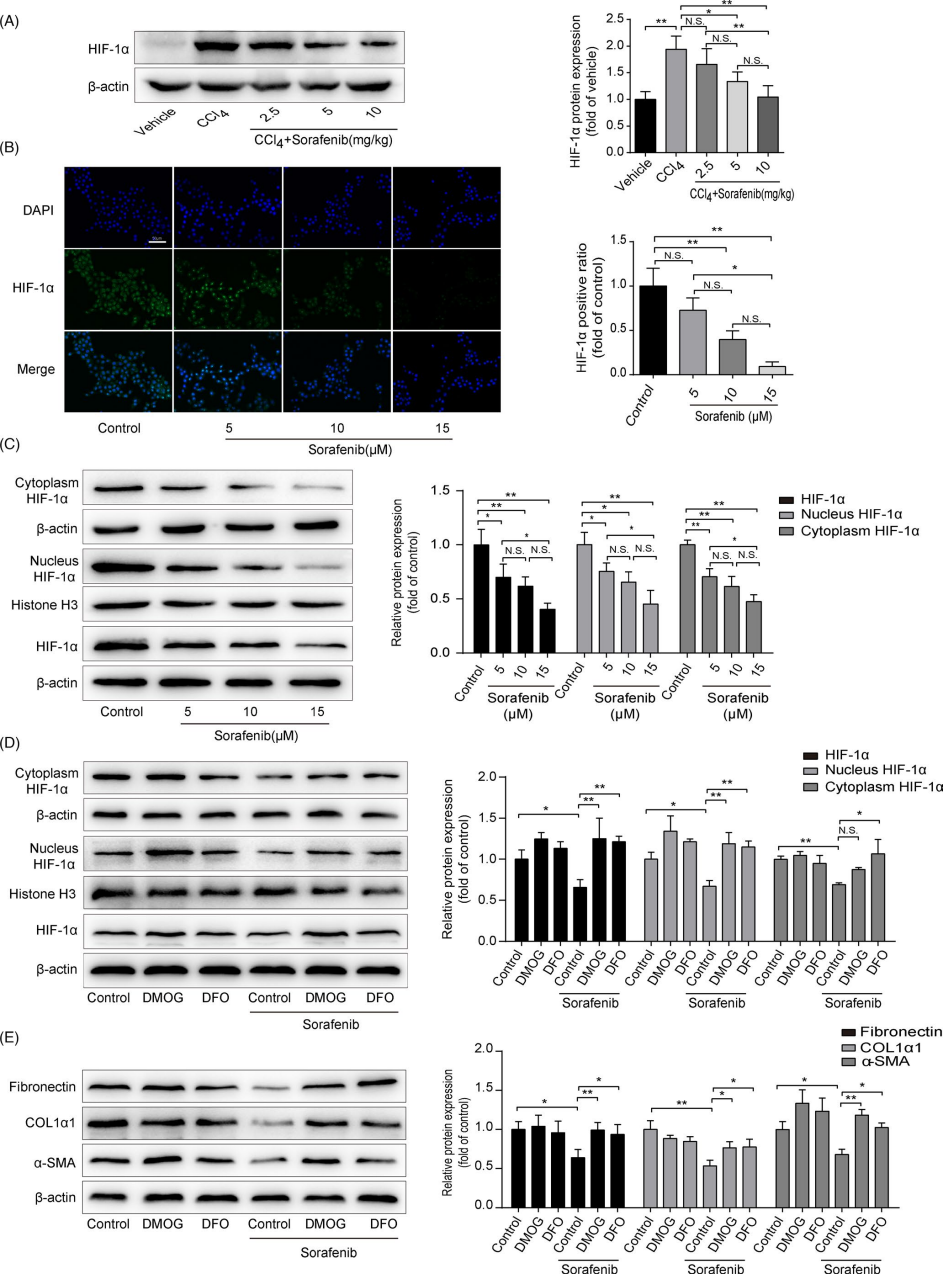
HIF‐1α expression is decreased during sorafenib‐induced anti‐fibrosis. (A) Western blot analysis of HIF‐1α protein in mouse tissues was performed. Data were presented as the mean ± SD of 3 independent experiments. *p < 0.05, **p < 0.01. N.S. not significant. HSC‐T6 cells were treated with sorafenib (5, 10, 15 μM) for 24 h. (B) HIF‐1α expression in HSC‐T6 cells was measured by immunofluorescence (Scale bar: 50 μm). Data were presented as the mean ± SD of 3 independent experiments. *p < 0.05, **p < 0.01. N.S. not significant. (C) Western blot analysis of HIF‐1α protein totally, in the cytoplasm and in the nucleus was performed, respectively. Data were presented as the mean ± SD of 3 independent experiments. *p < 0.05, **p < 0.01. N.S. not significant. HSC‐T6 cells were treated with DMOG (1 mM), DFO (100 μM) or/and sorafenib (10 μM) for 24 h. (D) Western blot analysis of HIF‐1α protein totally, in the cytoplasm and in the nucleus was performed, respectively. Data were presented as the mean ± SD of 3 independent experiments. *p < 0.05, **p < 0.01. N.S. not significant. (E) Western blot analyses of α‐SMA, COL1α1 and fibronectin proteins were performed. Data were presented as the mean ± SD of 3 independent experiments. *p < 0.05, **p < 0.01

It is interesting to note that DFO, a ferroptotic inhibitor, is also a HIF‐1α prolylhydroxylase inhibitor, which protects HIF‐1α protein from degradation.^29^ HIF‐1α protein level was significantly elevated in fibrotic liver compared to vehicle‐treated liver, which was reduced by sorafenib (Figure 5A). Furthermore, when exposed HSC‐T6 cells to sorafenib, fluorescence staining assay showed a decline of HIF‐1α both in cytoplasm and in nucleus (Figure 5B). Western blot analysis confirmed that HIF‐1α was decreased in sorafenib‐treated HSC‐T6 cells (Figure 5C). Considering that HIF‐1α is a transcription factor and acts its role in the nucleus mostly, the level of HIF‐1α protein in cytoplasm and nucleus of HSC‐T6 cells was detected respectively. As shown in Figure 5C, sorafenib decreased HIF‐1α expression both in the cytoplasm and in the nucleus.

To further explore the role of HIF‐1α in sorafenib‐induced anti‐fibrosis effect, two HIF‐1α prolylhydroxylase inhibitors, dimethyloxallyl glycine (DMOG) and DFO, were exposed to stabilize HIF‐1α protein in HSC‐T6 cells for 24 h. As illustrated in Figure 5D, HIF‐1α was significantly increased in the nucleus when treated with DMOG and DFO. In the cytoplasm, HIF‐1α level was increased in DFO‐treated cells, but not affected by DMOG (Figure 5D). Interestingly, DMOG and DFO also offset sorafenib anti‐fibrotic effect, as evidenced by α‐SMA, COL1α1 and fibronectin expressions (Figure 5E). Overall, these data demonstrate that sorafenib decreases HIF‐1α both in HSC nucleus and in cytoplasm. On the contrary, stabilization of HIF‐1α protein impedes anti‐fibrotic effect of sorafenib.

### 3.6 Sorafenib triggers HSC ferroptosis via HIF‐1α/SLC7A11‐dependent mechanism

**FIGURE 6:**
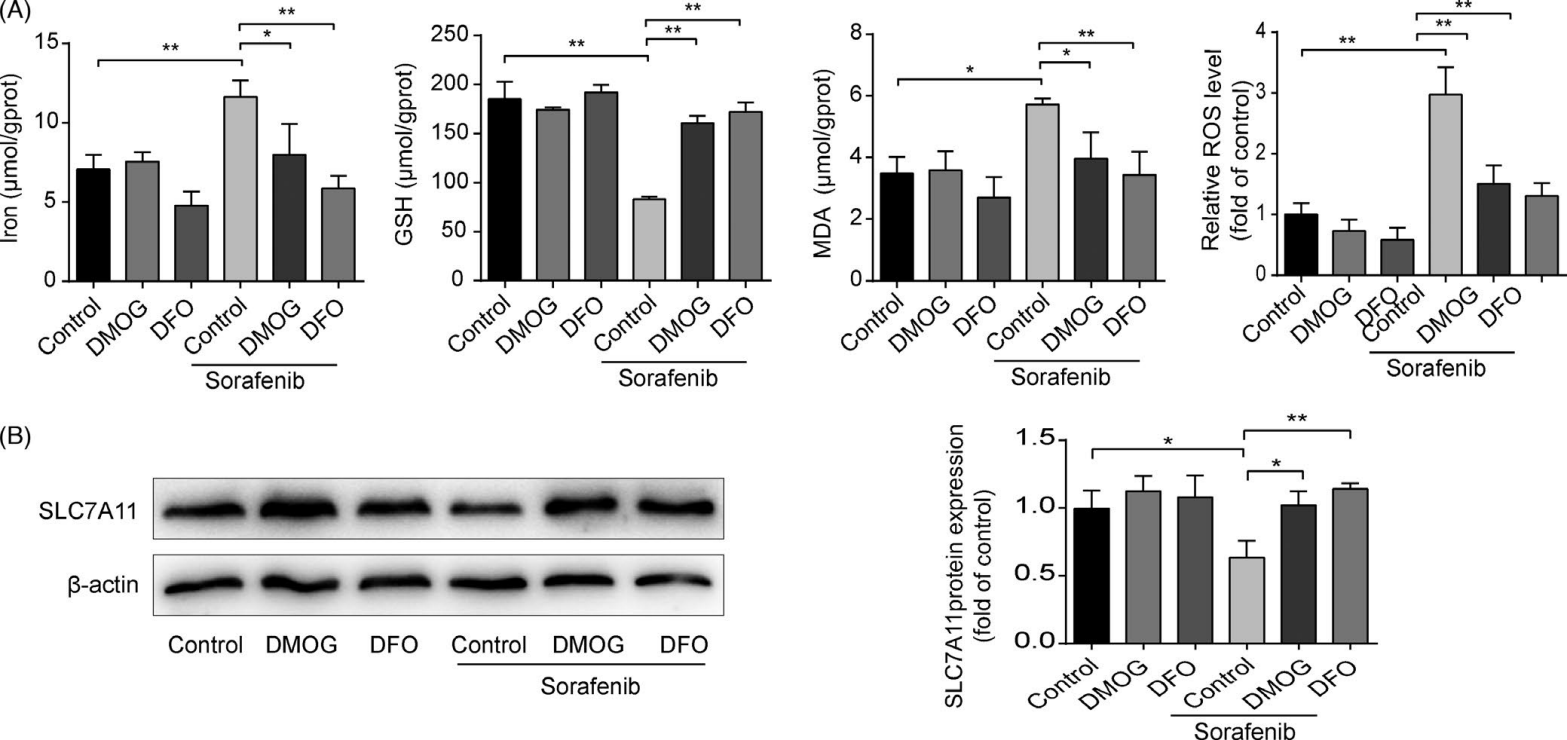
Stabilization of HIF‐1α protein abolishes HSC ferroptosis and SLC7A11 reduction induced by sorafenib. HSC‐T6 cells were treated with DMOG (1 mM), DFO (100 μM) or/and sorafenib (10 μM) for 24h. (A) Iron, MDA and GSH contents in cell lysates were measured by kits. Intracellular ROS generation was detected with DCFH‐DA probe. Data were presented as the mean ± SD of 3 independent experiments. *p < 0.05, **p < 0.01. (B) Western blot analysis SLC7A11 protein was performed. Data were presented as the mean ± SD of 3 independent experiments. *p < 0.05, **p < 0.01

**FIGURE 7:**
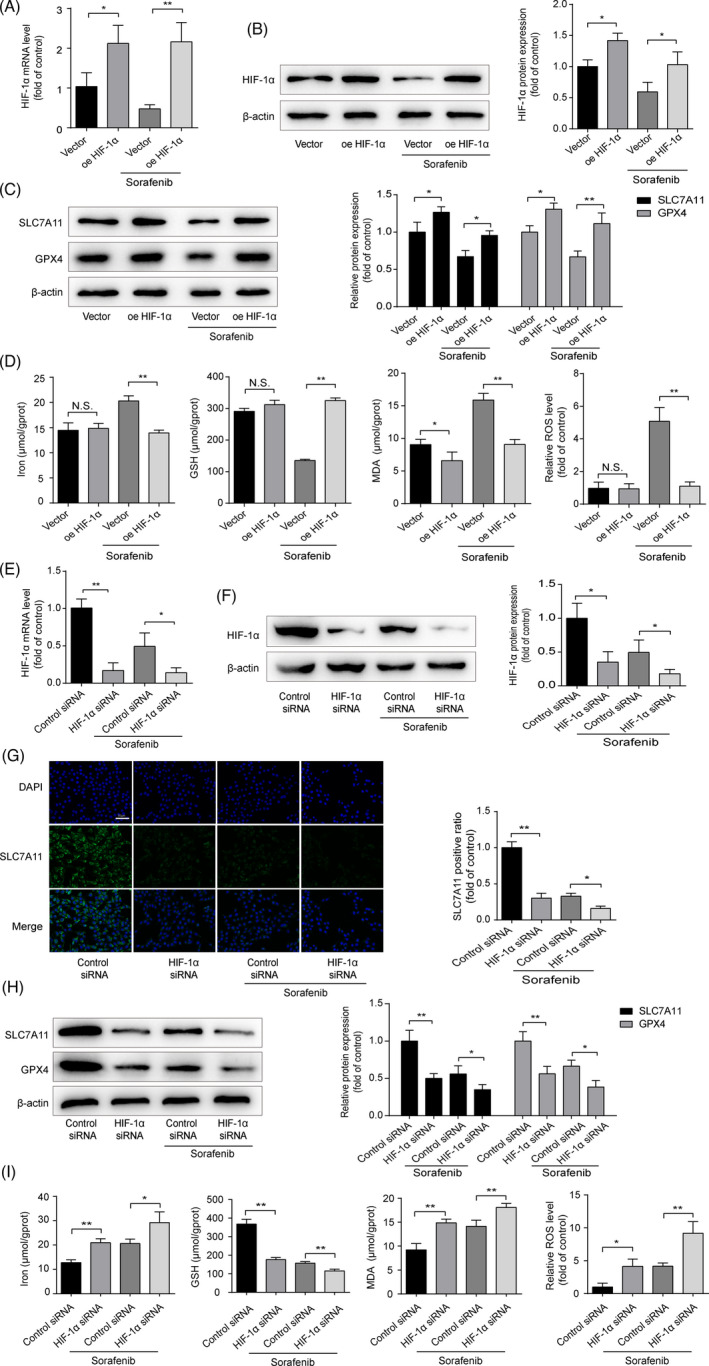
Sorafenib triggers HSC ferroptosis via HIF‐1α/SLC7A11‐dependent mechanism. Exposure HIF‐1α plasmid to HSC‐T6 cells for 48 h, with or without sorafenib (10 μM) treatment for 24 h. HIF‐1α (A) mRNA and (B) protein levels were measured by qRT‐PCR and Western blot. Data were presented as the mean ± SD of 3 independent experiments. *p < 0.05, **p < 0.01. (C) Western blot analyses of SLC7A11 and GPX4 proteins were performed. Data were presented as the mean ± SD of 3 independent experiments. *p < 0.05, **p < 0.01. (D) The contents of iron, MDA, and GSH in cell lysates were measured by kits. Intracellular ROS generation was detected with DCFH‐DA probe. Data were presented as the mean ± SD of 3 independent experiments. *p < 0.05, **p < 0.01. N.S. not significant. Exposure HIF‐1α siRNA to HSC‐T6 cells for 48 h, with or without sorafenib (10 μM) treatment for 24 h. HIF‐1α (E) mRNA and (F) protein levels were measured by qRT‐PCR and Western blot. Data were presented as the mean ± SD of 3 independent experiments. *p < 0.05, **p < 0.01. (G) The expression of SLC7A11 was measured by immunofluorescence (Scale bar: 50 μm). Data were presented as the mean ± SD of 3 independent experiments. *p < 0.05, **p < 0.01. (H) Western blot analyses of SLC7A11 and GPX4 proteins were performed. Data were presented as the mean ± SD of 3 independent experiments. *p < 0.05, **p < 0.01. (I) The contents of iron, MDA and GSH in cell lysates were measured by Kits. Intracellular ROS generation was detected with DCFH‐DA probe. Data were presented as the mean ± SD of 3 independent experiments. *p < 0.05, **p < 0.01

To gain insight into the potential signalling pathways in sorafenib‐triggered HSC ferroptosis, DFO and DMOG were applied to stabilize HIF‐1α protein. As shown in Figure 6A, sorafenib‐induced ferroptotic markers were mostly hindered by DFO and DMOG in HSC‐T6 cells. Similarly, protection of HIF‐1α from degradation could abrogated the depletion of SLC7A11 induced by sorafenib (Figure 6B). The data obtained with the pharmacological blockers were strengthened by the application of specific plasmid HIF‐1α. As shown in Figure 7A and B, HIF‐1α mRNA and protein levels were significantly increased in HSC‐T6 cells after 48‐h transfection. SLC7A11 and GPX4 proteins were significantly upregulated in HIF‐1α‐overexpressed HSC‐T6 cells (Figure 7C). Besides, sorafenib‐induced ferroptosis was curbed by HIF‐1α overexpression (Figure 7D). Taken together, overexpression of HIF‐1α elevates SLC7A11 protein level, which impairs sorafenib‐induced HSC ferroptosis.

**FIGURE 8:**
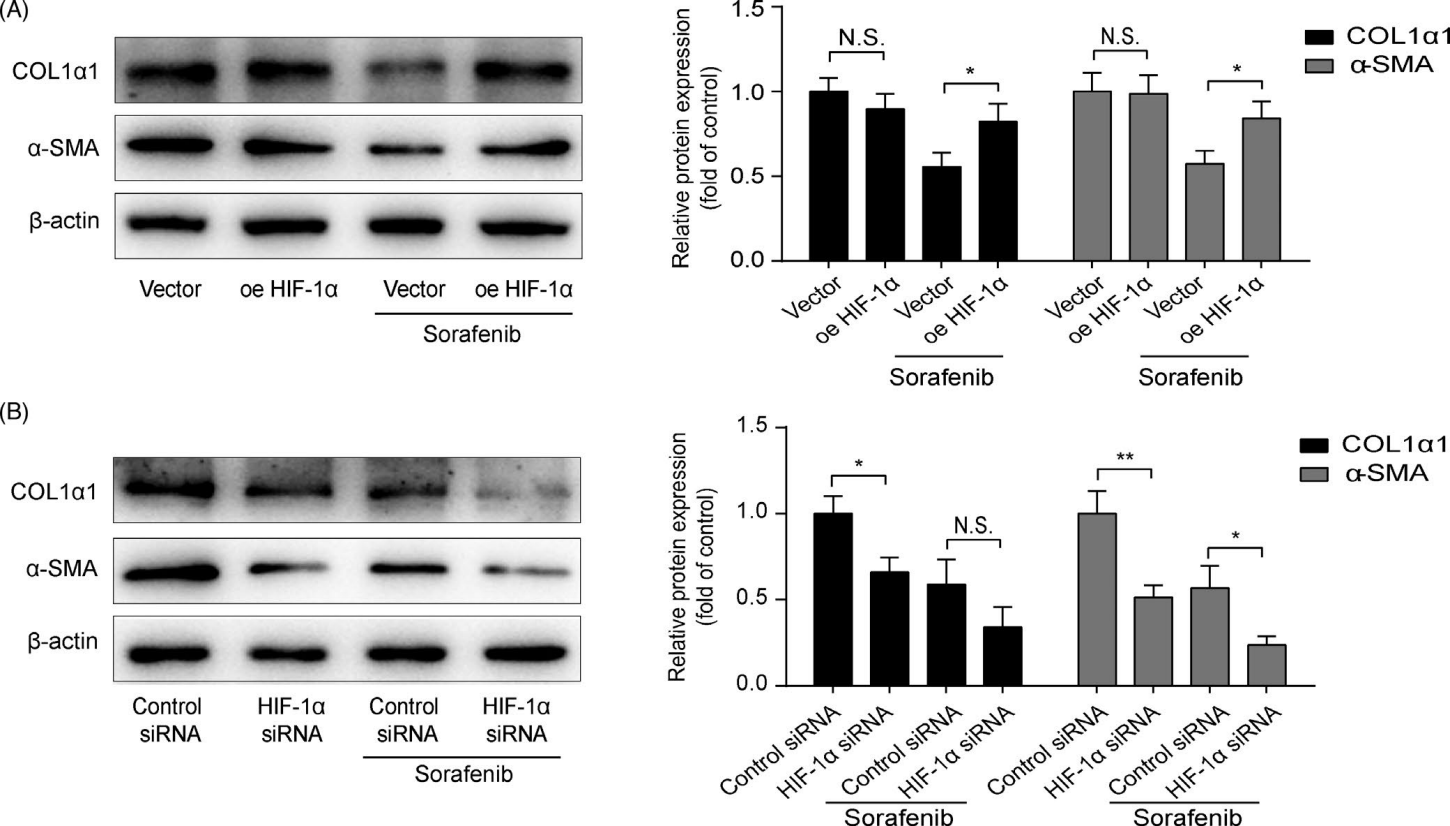
HIF‐1α is involved in the anti‐fibrotic effect of sorafenib. Western blot analyses of α‐SMA and COL1α1 proteins were performed in(A) HIF‐1α‐overexpressed and (B) HIF‐1α‐silenced HSC‐T6 cells, with or without sorafenib (10 μM) treatment for 24 h. Data were presented as the mean ± SD of 3 independent experiments. *p < 0.05, **p < 0.01. N.S. not significant

To further establish the vital role of HIF‐1α/SLC7A11 in HSC ferroptosis, siRNA specific for rat HIF‐1α (HIF‐1α siRNA) was used to knockdown the HIF‐1α expression. As illustrated in Figure S2, 7E and F, HIF‐1α siRNA‐I induced a significant decrease in HIF‐1α mRNA and protein levels in HSC‐T6 cells after transfection for 48 h, in comparison with the cells transfected with control siRNA. Fluorescence intensity of SLC7A11 was notably decreased by HIF‐1α siRNA with or without sorafenib treatment (Figure 7G). GPX4 and SLC7A11 protein levels significantly reduced in HIF‐1α‐silenced HSC‐T6 cells (Figure 7H). In addition, depletion of HIF‐1α strengthened ferroptosis induced by sorafenib (Figure 7I). Finally, ECM accumulation was also determined. Low expressions of α‐SMA and Col1α1 in sorafenib‐treated HSC‐T6 cells were reinforced by silence of HIF‐1α and were abated partly by overexpression of HIF‐1α (Figure 8A and B). Based on the above data, we prove that HIF‐1α/SLC7A11 is involved in sorafenib‐induced HSC ferroptosis, and silencing HIF‐1α can intensify the anti‐fibrotic effect of sorafenib.

## 4 DISCUSSION

**FIGURE 9:**
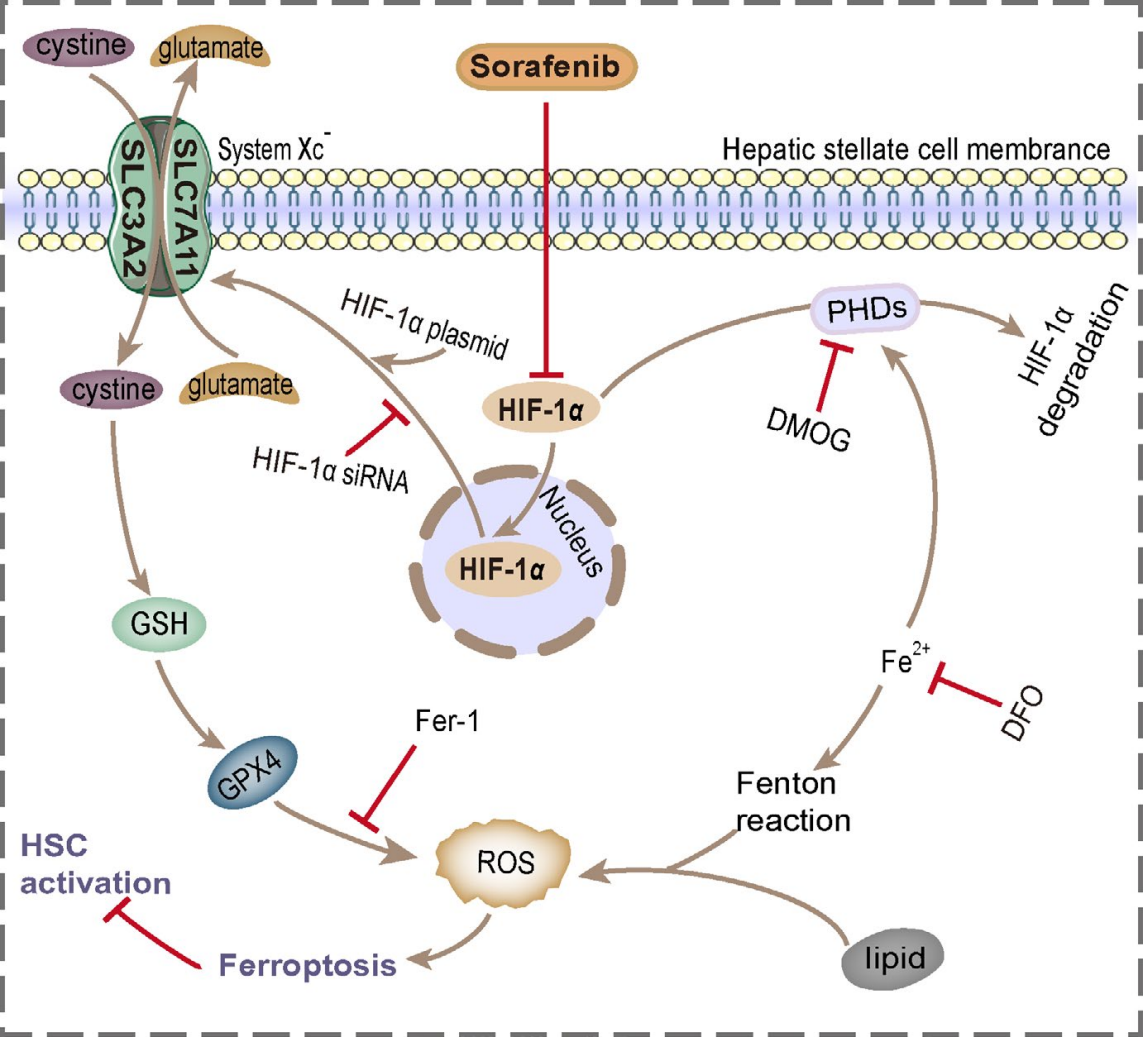
Sorafenib ameliorates liver fibrosis by triggering ferroptosis in HSC via HIF‐1α/SLC7A11 pathway. Treatment with sorafenib induces a decrease in HIF‐1α, which in turn reduces SLC7A11 expression in HSCs, then leads to GPX4, GSH depletion and ROS excess, and ultimately induces HSC ferroptosis and ECM reduction

HSC acts a critical role in pathogenesis of liver fibrosis. In response to injury, HSCs differentiate into activated myofibroblasts, synthesize and deposit fibrillar collagen, which result in significant fibrosis. Therefore, inhibition of HSC is considered as a key strategy for therapeutic intervention of liver fibrosis.^5^, ^6^, ^7^ Ferroptosis, a new type of programmed cell death, has been identified as a therapeutic target in liver fibrotic diseases.^10^, ^11^ In this study, we provide evidence that sorafenib ameliorates CCl_4_‐induced liver fibrosis by triggering ferroptosis in HSCs through HIF‐1α/SLC7A11 pathway. Strikingly, inhibition of HSC ferroptosis hampers the anti‐fibrotic effect of sorafenib (Figure 9).

Sorafenib has been proved to be a potential drug for treating liver fibrosis. In hepatotoxic and cholestatic animal models, sorafenib remarkably alleviated liver injury and decrease ECM deposition.^30^ In vitro, sorafenib decreased cell viability in a dose‐ and time‐dependent manner in both HSC cell lines and primary HSCs. Besides, sorafenib exhibited a proliferative inhibition and apoptotic promotion of HSCs.^14^ In our study, different inhibitors of cell death were used to explore the mechanism of sorafenib on HSC death mode. Both ferroptotic inhibitors (Fer‐1 and DFO) and apoptotic inhibitor (ZVAD‐FMK) could reverse the decreased HSC viability in sorafenib‐treated group. Attractively, Fer‐1 and DFO demonstrated a higher ability to impede the inhibition role of sorafenib in HSCs than ZVAD‐FMK. In addition, reduced mitochondrial volume, condensed membrane density, broke mitochondrial cristae and elevated ROS level were observed in sorafenib‐treated HSCs. Above phenomena are considered as parts of ferroptotic events.^8^, ^9^ Intriguing, the impairment of mitochondrial function and elevated ROS level are showed in apoptotic cells as well.^31^, ^32^ Unfortunately, we have not found an accurate analytical method to distinguish them, we only explored the mechanism of sorafenib anti‐fibrotic effect with focus on HSC ferroptosis.

SLC7A11 is a main structural component of cell system Xc^−^, which participates in maintaining redox homeostasis. Via system Xc^−^, cystine is internalized by the cells and then synthesizes cysteine and GSH. GPX4, an important antioxidant enzyme, maintains the balance of ROS. GPX4 is constitutively expressed in various cells, including hepatocytes.^33^, ^34^ SLC7A11 is less expressed in the ordinary liver, while can be found in liver injury (eg drug‐induced injury and toxin‐induced injury).^35^, ^36^ In our study, IHC staining showed localization of SLC7A11 and GPX4 in CCl_4_‐treated mouse hepatocytes (Figure S1). Likewise, SLC7A11 and GPX4 were expressed in mouse primary HSCs.^13^, ^37^ More importantly, it has been found that inhibitions of SLC7A11 and GPX4 are involved in cell ferroptosis.^9^, ^38^ Here, we focused on SLC7A11 and GPX4 expressions in HSCs around fibrotic scars. In the CCl_4_‐treated group, SLC7A11 and GPX4 were significantly expressed around the scar, while decreased with sorafenib treatment (Figure S1). These suggest that the anti‐fibrotic process of sorafenib is accompanied by ferroptosis in HSCs.

Inducing HSC ferroptosis provides a new way for the treatment of liver fibrosis, while ferroptotic inducers can also trigger some other cells resided in the liver and finally may cause some side effects. Wang et al. found that ferroptosis occurs in hepatocytes and macrophages, which is hazardous to the recovery of liver injury.^17^ Therefore, drug design aims at induction of ferroptosis in HSCs rather than in hepatocytes and macrophages. Our data showed that sorafenib (10 μM) induces ferroptosis in HSCs, with less effect on hepatocytes and macrophages. Indeed, GSH level, a major cellular defence system against ROS, is higher in hepatocytes than in HSCs.^39^ That may be why hepatocytes are more resistant to sorafenib‐induced ferroptosis than HSCs. In addition, the main function of macrophages is to engulf harmful microorganisms and destroy them, and these processes are largely dependent on the production of ROS.^40^ Thus, macrophages may be able to sense the invasion of sorafenib and achieve coordinated ROS production and clearance, ultimately resist ferroptosis.

In this study, we found sorafenib‐induced ferroptosis in HSCs accompanied by reduced SLC7A11 and HIF‐1α proteins. Moreover, silencing HIF‐1α decreased SLC7A11 protein level, and overexpression of HIF‐1α with plasmid or stabilizers increased SLC7A11 protein level. Decreased HIF‐1α and SLC7A11 in HSC‐T6 cells strengthened sorafenib‐induced cell ferroptosis and ECM reduction. On the contrary, increased expressions of HIF‐1α and SLC7A11 inhibited HSC ferroptosis and impaired the sorafenib anti‐fibrotic effect. It may be conceivable that sorafenib induces HSC ferroptosis, at least in part, via HIF‐1α/SLC7A11.

PTGS2, also known as cyclooxygenase 2 (COX2), is a potent enzyme that initiates inflammation and promotes prostaglandin synthesis following stimulation by various inflammatory factors.^41^, ^42^ Recently, gene networks analysed by bioinformatics have shown that PTGS2 is the hub gene in the biology of cell ferroptosis.^43^ As well, PTGS2 expression is increased during ferroptosis in various cells, such as vascular smooth muscle cells, neuronal cells and HSCs.^44^, ^45^, ^46^

Consistent with the previous studies, PTGS2 expression was markedly increased both in sorafenib‐treated fibrotic livers and in HSC‐T6 cells, which was accompanied by ferroptotic events. However, PTGS2 expression w

as not affected by HIF‐1α with or without sorafenib treatment (Figure S3A, B, C and D). In other words, sorafenib elevating PTGS2 may be not through HIF‐1α/SLC7A11 pathway, and the underlying mechanism needs to be further explored.

In summary, our findings in the present study demonstrate that HSC ferroptosis acts a vital role in sorafenib anti‐fibrosis. And sorafenib induces HSC ferroptosis, at least in part, via HIF‐1α/SLC7A11 pathway. Blockade of ferroptosis with inhibitors offsets the reduction of ECM in liver. This study clearly demonstrates that sorafenib induces ferroptosis in HSCs, and these findings will provide new targets for the treatment of liver fibrosis.

## CONFLICT OF INTEREST

The authors declare no conflict of interests.

## AUTHOR CONTRIBUTIONS

LF, SY and CW designed the study. SY performed the main experiments, GL, LZ, JL, LL and SC participated in some experiments. CW and SY drafted the manuscript, and LF revised the manuscript. All authors provided final approval and agree to be accountable for all aspects of the work.

## Supporting information

Fig S1

Fig S2

Fig S3

Supplementary Material

## Data Availability

Data supporting the results of this study can be obtained from the corresponding author upon reasonable request.
