# Supplementary figures and images for: Sorafenib attenuates liver fibrosis by triggering hepatic stellate cell ferroptosis via HIF‐1α/SLC7A11 pathway
Source: Cell Prolif. 2021 Nov 22;55(1):e13158. doi: 10.1111/cpr.13158 (PMC8780895; doi:10.1111/cpr.13158)

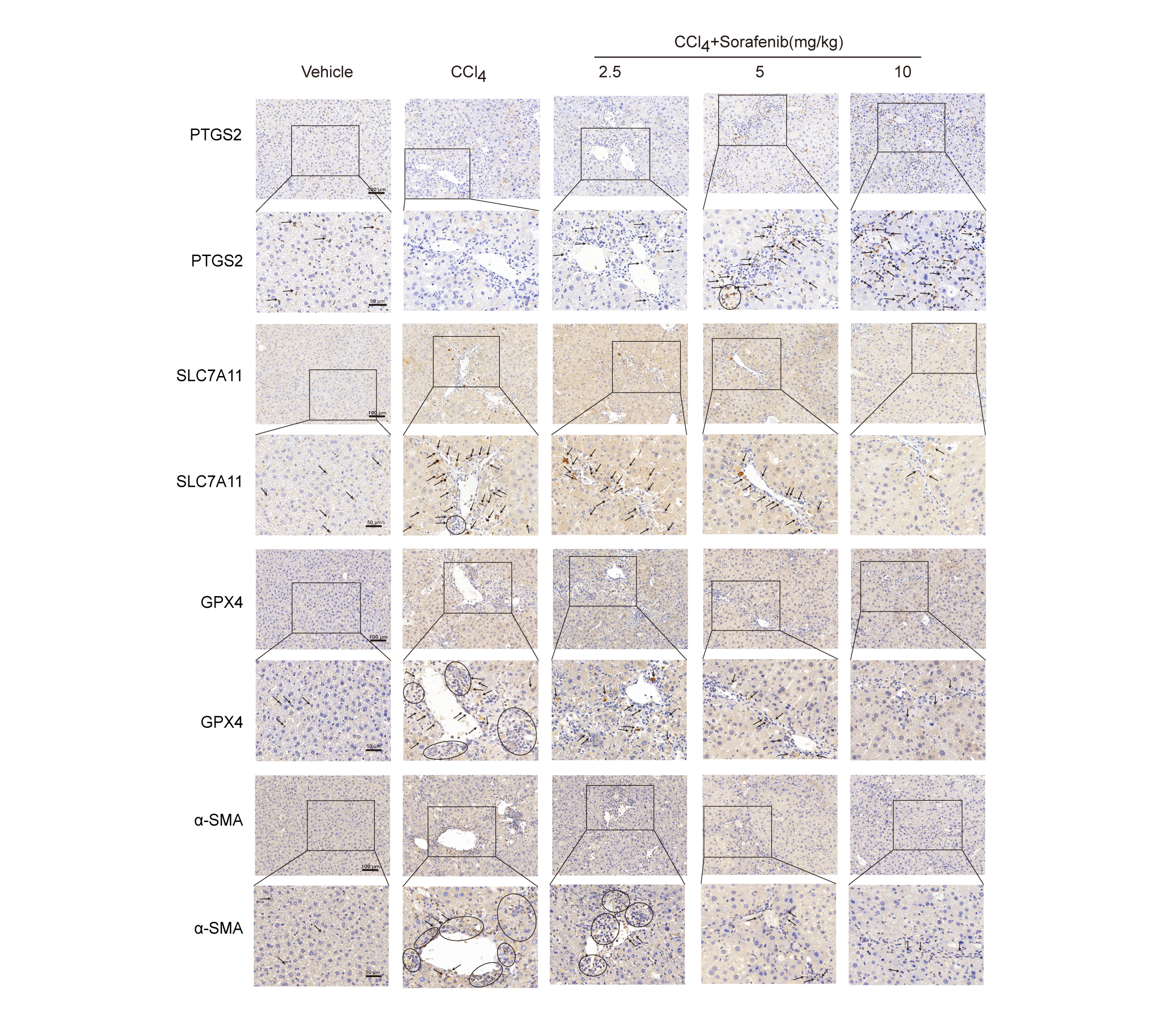

Supplement: Supplementary file 1 — Fig S1 [file CPR-55-e13158-s003.tif]

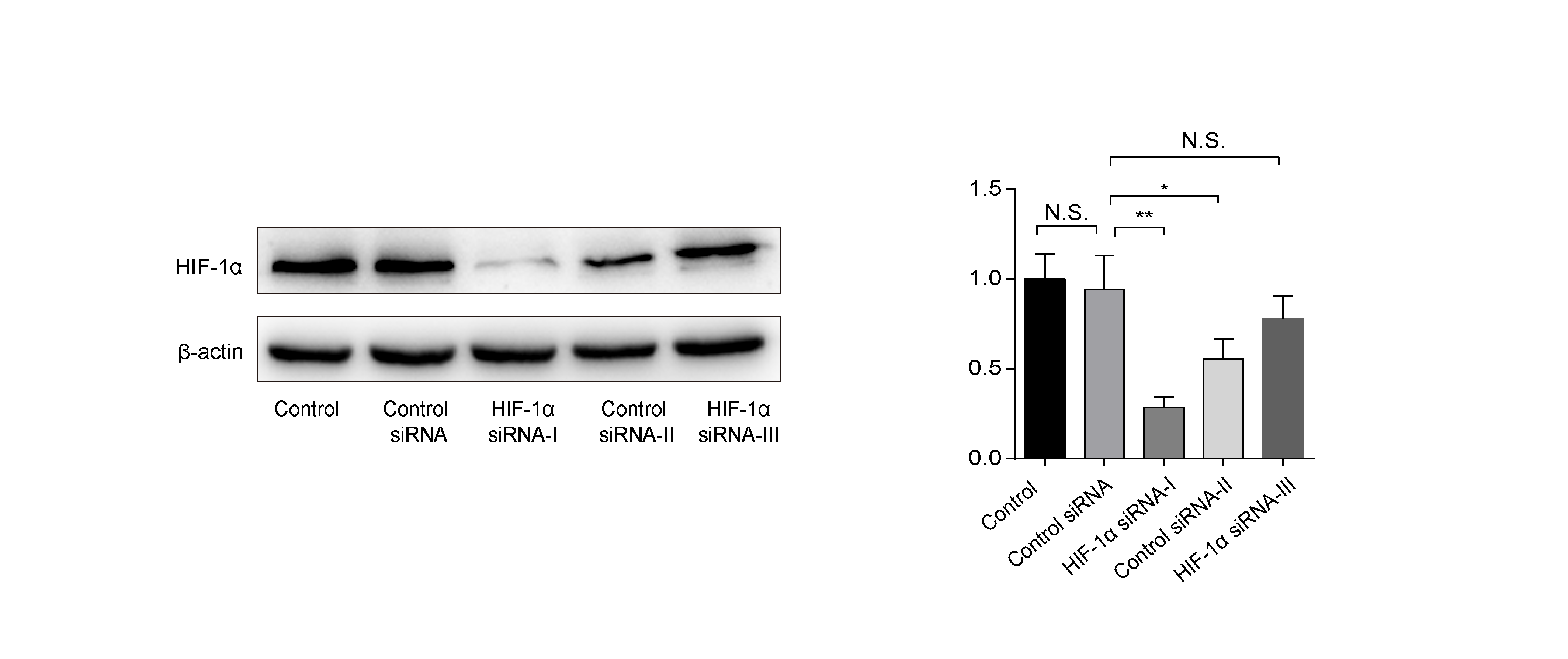

Supplement: Supplementary file 2 — Fig S2 [file CPR-55-e13158-s001.tif]

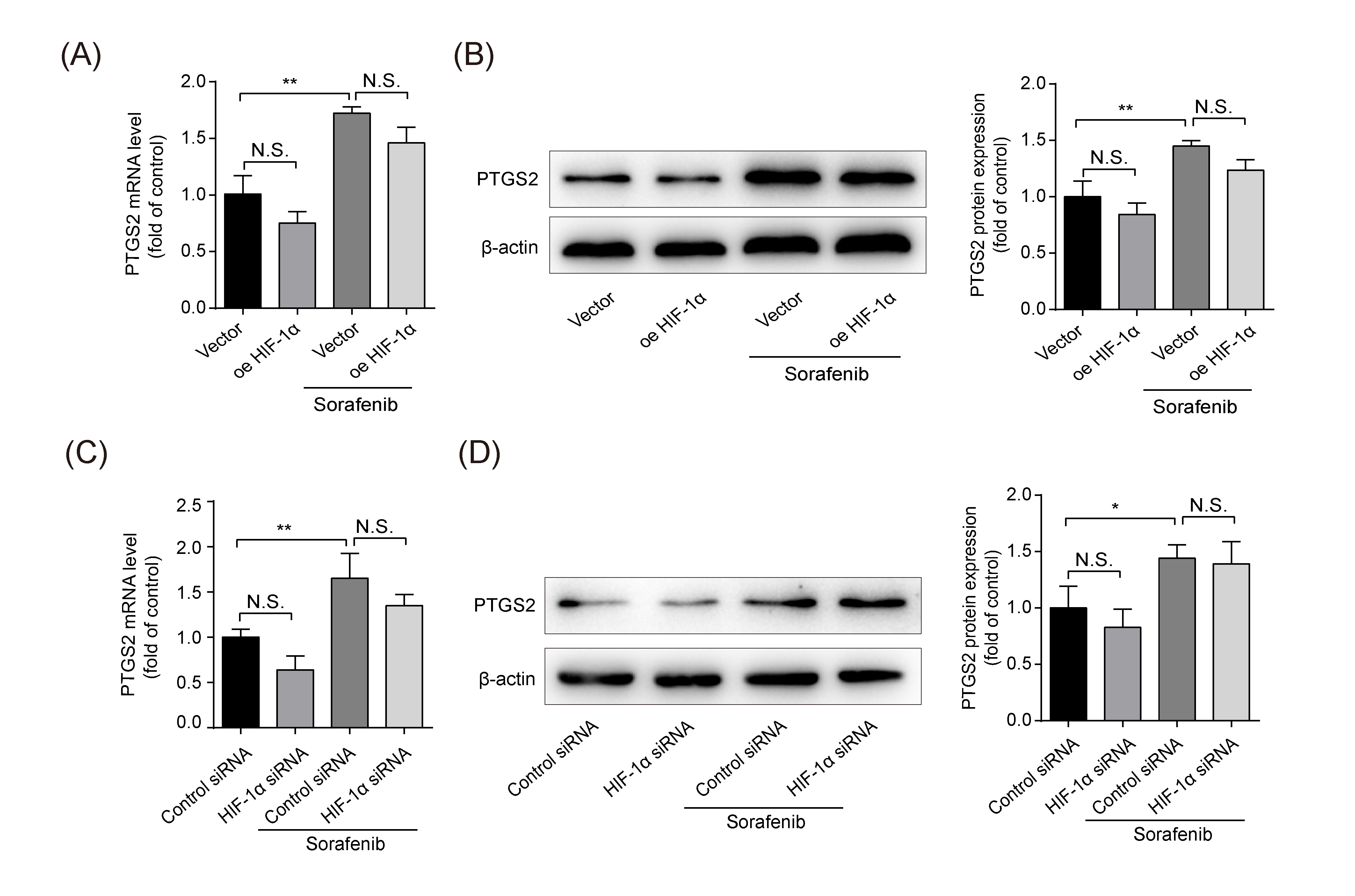

Supplement: Supplementary file 3 — Fig S3 [file CPR-55-e13158-s004.tif]
