# Supplementary material for: Sorafenib attenuates liver fibrosis by triggering hepatic stellate cell ferroptosis via HIF‐1α/SLC7A11 pathway
Source: Cell Prolif. 2021 Nov 22;55(1):e13158. doi: 10.1111/cpr.13158 (PMC8780895; doi:10.1111/cpr.13158)
Supplement: Supplementary file 4 — Supplementary Material [file CPR-55-e13158-s002.docx]

**SUPPLEMENTARY FIGURE LEGENDS**

**FIGURE S1** PTGS2, SLC7A11, GPX4 and α-SMA expressions in mice liver were detected via immunohistochemistry (Scale bar: 100 μm). The magnified images showed areas of liver fibrosis scarring. (Scale bar: 50 μm) (n=3 in every group). Black arrows and black circles indicated PTGS2, SLC7A11, GPX4 and α-SMA-positive cells.

**FIGURE S2** Three HIF-1α siRNAs were exposed to HSC-T6 cells for 48 h. Western blot analysis of HIF-1α protein was performed. Data were presented as the mean ± SD of 3 independent experiments. **P* < 0.05, ***P* < 0.01, N.S. not significant.

**FIGURE S3** PTGS2 is not affected by HIF-1α/SLC7A11 axis. Exposure HIF-1α plasmid to HSC-T6 cells for 48 h, with or without sorafenib (10 μM) treatment for 24 h. PTGS2 (A) mRNA and (B) protein levels were performed with qRT-PCR and western blot. Data were presented as the mean ± SD of 3 independent experiments. **P* < 0.05, ***P* < 0.01, N.S. not significant. Exposure HIF-1α siRNA to HSC-T6 cells for 48 h, with or without sorafenib (10 μM) treatment for 24 h. PTGS2 (C) mRNA and (D) protein levels were performed with qRT-PCR and western blot. Data were presented as the mean ± SD of 3 independent experiments. **P* < 0.05, ***P* < 0.01, N.S. not significant.
